# Predictive Value of FDG Uptake on PET for Future Immune Checkpoint Inhibitor-Mediated Colitis: A Case Series

**DOI:** 10.3390/jcm14010256

**Published:** 2025-01-04

**Authors:** Malek Shatila, Kei Takigawa, Yang Lu, Andres Caleb Urias Rivera, Nitish Mittal, Abdullah Sagar Aleem, Sean Ngo, Eric Lu, Deanna Wu, Gabriel Sperling, Sidra Naz, Bryan Schneider, Anusha Shirwaikar Thomas, Yinghong Wang

**Affiliations:** 1 Department of Gastroenterology, Hepatology & Nutrition, The University of Texas MD Anderson Cancer Center, Houston, TX 77030, USA; hw8704@wayne.edu (M.S.); ehl2@tamu.edu (E.L.); deanna.w@wustl.edu (D.W.); snaz@mdanderson.org (S.N.); asthomas1@mdanderson.org (A.S.T.); 2 Department of Internal Medicine, Baylor College of Medicine, Houston, TX 77030, USA; kei.takigawa@bcm.edu (K.T.); andres.uriasrivera@bcm.edu (A.C.U.R.); 3 Department of Nuclear Medicine, The University of Texas MD Anderson Cancer Center, Houston, TX 77030, USA; ylu10@mdanderson.org; 4 Department of Internal Medicine, University of Texas Health Science Center, Houston, TX 77030, USA; nitish.mittal@uth.tmc.edu (N.M.); abdullah.aleem@uth.tmc.edu (A.S.A.); sean.b.ngo@uth.tmc.edu (S.N.); 5 Department of Internal Medicine, University of Texas Medical Branch, Galveston, TX 77555, USA; gabriel.sperling@unchealth.unc.edu; 6 Department of Thoracic Medical Oncology, University of Michigan, Ann Harbor, MI 48109, USA; bryansch@med.umich.edu

**Keywords:** immune-mediated colitis (IMC), immune checkpoint inhibitors (ICI), PET/CT imaging, surveillance, diarrhea

## Abstract

**Objectives**: Immune-mediated colitis (IMC) is a common immune-related adverse event during immune checkpoint inhibitor (ICI) therapy. This case series and review aimed to highlight atypical cases of IMC and explore the potential of PET/CT to predict imminent ICI colitis. **Methods**: Through a descriptive, retrospective study at a tertiary cancer center, we identified adult patients receiving ICIs for any cancer between 2010 and 2022 who also underwent PET/CT for routine cancer surveillance during this time. We included patients who had signs and symptoms of colitis and reviewed their surveillance PET/CT scans obtained 2 to 6 weeks before and up to 3 months after diagnosis. **Results**: For the 33 included patients, surveillance scans were reviewed in collaboration with a nuclear radiologist. A total of 17 patients (51.5%) received combination therapy, while 14 (42.4%) received anti–PD-1/PD-L1 monotherapy. While ICI therapy has a median duration of 6.5 months, most patients (72.7%) had negative surveillance PET/CT for colitis. Diarrhea and colitis severity were similar among those with positive and negative findings for colitis on surveillance PET/CT. The outcomes of colitis were similar, with an 81.8% resolution in patients with negative PET/CT and 71.4% in patients with positive PET/CT. **Conclusions**: PET/CT imaging did not appear to assist in predicting IMC. This may be due to the long interval between clinical IMC and surveillance PET/CT imaging. The continued use of clinical criteria combined with laboratory markers, e.g., lactoferrin and calprotectin, and endoscopy/histology will enable more accurate detection and timely treatment of IMC.

## 1. Introduction

Immune checkpoint inhibitors (ICIs) are an increasingly used form of cancer treatment and have been approved for a growing number of malignancies [1]. They act as monoclonal antibodies to important proteins that regulate immune cell proliferation, such as programmed cell death protein-1/ligand-1 (PD-1/L1) and cytotoxic T-lymphocyte–associated protein-4 (CTLA-4), to provoke an enhanced immune response. This stimulation of the immune system can give rise to immune-related adverse events (irAEs) in various organs, with immune-mediated colitis (IMC) being the most common and severe of these toxicities [2].

The initial evaluation of IMC relies heavily on clinical history and the results of stool infectious and inflammatory work-ups, with endoscopic biopsy being the ultimate means of diagnosis [3]. Imaging modalities such as computed tomography (CT) of the abdomen have traditionally been the cornerstone of working up nonspecific abdominal pain or any suspicion of colitis, but there is limited evidence to support their use in IMC [4]. The few published studies on the subject showed a low sensitivity for CT to detect IMC [5,6]. Other studies have explored the utility of a routine study for cancer staging, fludeoxyglucose-18 (FDG) positron emission tomography (PET), in evaluating colitis [7]. PET scans are typically conducted every 3 months in patients with advanced malignancy and may show increased glucose uptake in areas of active inflammation [8]. To date, only one study has explored the use of PET in the evaluation of IMC, showing a significant correlation between PET findings and clinical symptoms [9]. That said, no study to date has investigated the role of PET findings suggestive of subclinical inflammation prior to the onset of colitis in predicting future colitis, nor has the meaningfulness of the resolution of PET changes after colitis treatment been explored [4,6,7,10,11].

Given the paucity of data on the subject, this study aimed to explore the utility of routine PET scans for cancer staging in predicting impending IMC as well as evaluating IMC treatment response. Specifically, we present a descriptive comparison of PET/CT findings and IMC clinical features to highlight the variability of disease manifestations and the limited role of PET findings.

## 2. Materials and Methods

This was a descriptive, retrospective study at a tertiary care cancer center that included a random sample of adult patients receiving ICIs for any cancer type between September 2010 and January 2022 who developed IMC and also underwent PET/CT for routine cancer surveillance during this time. Only surveillance PET/CT scans that occurred between 2 and 6 weeks before the initial colitis diagnosis and up to 3 months after diagnosis were used. These were reviewed by a collaborating radiologist. Initially, all patients were planned to be included in this study, with an additional control group without colitis included for adequate comparison. However, based on radiologist availability, it was decided post hoc to include a random sample of 33 patients with colitis in the final study. Patients with other etiologies of colitis, including infectious, autoimmune, and radiation-related causes, were excluded. Patients receiving metformin at the time of PET were also excluded to avoid the confounding effect of metformin on increasing FDG uptake in the colon on PET. All patient data were obtained through institutional electronic health records. We collected patient demographic information including age, sex, and race/ethnicity; cancer type; ICI treatment details; and mortality data. We also collected IMC-related clinical information. This study was approved by the institutional review board (PA18-0472) in 2018 with a waiver of patients’ informed consent.

### 2.1. IMC Screening and Clinical Information

Patients’ medical records were initially screened for IMC based on the presence of stool studies. Eligible patients were then screened by independent reviewers and included after manual chart review. Colitis-related variables were collected, which included date of onset, peak severity as measured by the Common Terminology Criteria for Adverse Events (CTCAE) version 5, treatments, and outcomes such as recurrence, hospitalization, and ICI resumption.

### 2.2. Identification of Colon FDG Uptake on PET/CT

An experienced nuclear radiologist manually reviewed the imaging findings of PET/CTs performed within 2 to 6 weeks before the onset of colitis and up to 3 months after the onset of colitis. These dates were intentionally chosen: the 2- to 6-week cut-off was selected to assess PET’s ability to acutely predict the onset of IMC, and the 3-month cut-off was chosen to give time for the inflamed colon to heal after treatment. Only the PET/CT closest to the colitis diagnosis was reviewed in both cases. A positive PET finding for colitis was defined as the presence of diffuse, intense FDG uptake in the colon with associated wall thickening and adjacent fat stranding [12,13,14].

### 2.3. Data Analysis

The data were analyzed using SPSS 26.0. Categorical variables were described using the frequency and percentage of the total. Continuous variables were described by their median and interquartile range. The Mann–Whitney U nonparametric test was used to compare differences in medians between two groups of continuous variables. Further statistical analyses were not conducted due to limitations in sample size.

## 3. Results

### 3.1. Patient Characteristics

A total of 33 patients were included. The patients’ characteristics are summarized in Table 1. Our sample was mostly white (29, 87.9%) and female (20, 60.6%), with a median age of 62 (IQR: 52–76). Lung cancer was the most common cancer type (14, 46.7%), followed by melanoma (8, 26.7%). A total of 17 (51.5%) patients received combination immunotherapy, while 14 (42.4%) received anti-PD-1/L1 and 2 (6.1%) received anti-CTLA-4 therapy. The median duration of follow-up was 0.9 years, and the all-cause mortality rate was 59.4%.

### 3.2. Colitis-Related Characteristics

Of the 33 patients, 24 (72.7%) had colitis-negative PET/CT findings before IMC diagnosis compared to 9 (27.2%) with a positive PET/CT. Most patients had grade 2 or higher diarrhea and colitis (Table 2). Patients in both groups had similar rates of supportive and steroid treatments, but selective immunosuppressive therapy (SIT) use was less frequent among the negative PET/CT group (34.8%) compared to the positive PET/CT group (66.7%).

### 3.3. Clinical Outcomes

Colitis resolution rates were similar across both groups (Table 2). Interestingly, 60.9% of patients with negative PET/CT were hospitalized for IMC, and 43.5% were able to resume immunotherapy. Conversely, 44.4% of patients with positive PET/CT were hospitalized for IMC, and 62.5% were able to resume immunotherapy. Finally, a higher percentage of patients with positive PET/CT prior to colitis had persistent glucose uptake on PET/CT after colitis treatment (57.1%) compared to patients with a negative PET/CT (25.0%).

## 4. Representative Cases

### 4.1. Case #1

A 45-year-old White woman was initially diagnosed with adenocarcinoma of the lung. She was initially treated with radiation therapy and within 2 months began combination nivolumab/ipilimumab treatment, which led to CTCAE grade 2 diarrhea after 2 doses. PET/CT for staging was captured prior to the initiation of ICI therapy and was negative for colitis. She was treated empirically for IMC with 50 mg of prednisone daily with no improvement. Subsequently, she tested positive for Clostridioides difficile via DNA testing, which prompted oral vancomycin treatment for 10 days. However, her diarrhea persisted at similar severity. Additional laboratory testing showed positive stool lactoferrin and calprotectin levels of 46.1 mcg/g (normal < 50 mcg/g) after the clearance of *C. difficile*.

**Figure 1:**
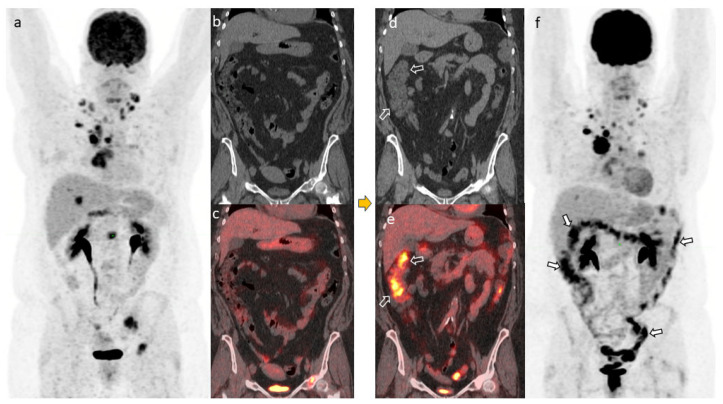
PET/CT scans in Case 1 prior to *Clostridioides difficile* diagnosis. The maximum intensity projection (MIP) image (**a**), coronal CT (**b**), and coronal PET/CT (**c**) showed no appreciable abnormal uptake in the colon. The PET/CT scans 1 month after treatment of diagnosed *C. difficile*. The coronal CT (**d**), coronal PET/CT (**e**), and MIP image (**f**) showed diffuse FDG uptake in the colon but without corresponding wall thickening on CT (arrows in (**d**–**f**)). This type of uptake was deemed nonspecific stimulation/inflammation from immunotherapy but was not severe enough to meet the diagnosis of colitis.

**Figure 2:**
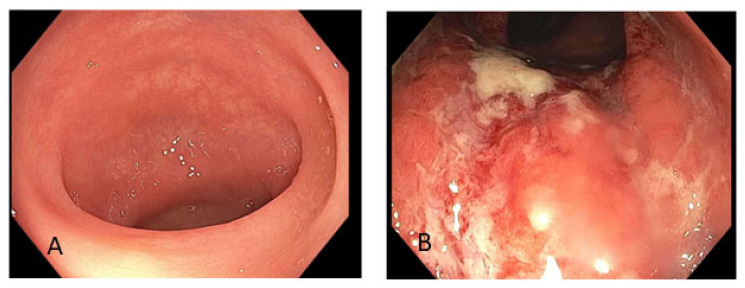
Endoscopic images. Normal appearing colon from Case 1 (**A**). Edema, erythema, and ulceration from Case 2 (**B**).

One month following the initial diarrhea symptoms, a PET/CT scan was obtained for re-staging. In addition to persistent metastatic tumor burden, it showed increased metabolic activity throughout the colon. The radiologist’s report indicated that this may have been due to physiologic activity (Figure 1). Within 1 month of the PET/CT, the patient underwent a colonoscopy, which showed a normal ileum and colon (Figure 2A) with pathology consistent of lymphocytic colitis. She was then given vedolizumab for a total of 3 doses and then missed follow-up until her death from cancer progression was reported 6 months later in the chart.

### 4.2. Case #2

A 54-year-old woman presented with metastatic carcinoma of the left breast. At that time, she underwent lumpectomy of the left breast and then received combination therapy with paclitaxel, trastuzumab, and pertuzumab. However, her cancer progressed despite these treatments and other regimens, and ultimately, she began treatment with nivolumab and a PI3K inhibitor due to progressive disease. This regimen was later switched to pan-AKT inhibitor therapy due to her refractory cancer status.

**Figure 3:**
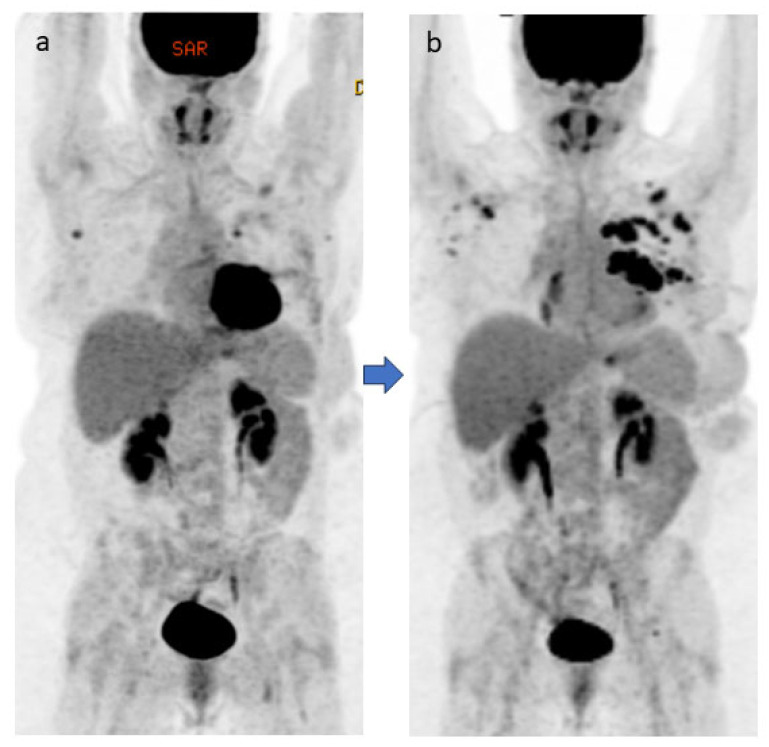
A female patient with metastatic left breast carcinoma and receiving immunotherapy. Neither the PET/CT completed 1 month before the colitis episode (maximum intensity projection [MIP] image (**a**)) nor the one completed 1 month after the colitis episode (MIP image (**b**)) showed any appreciable abnormal increased uptake in the colon.

Following two cycles of pan-AKT inhibitor therapy, the patient developed grade 3 diarrhea, which did not respond to loperamide; fever (maximum temperature 38.2 °C); and fatigue, which led to a hospital admission 2 years after her initial diagnosis. Despite negative findings on the infection work-up, the patient was given oral vancomycin and cefepime empirically because of the diarrhea progression. A flexible sigmoidoscopy during the hospital admission revealed edema, erythema, and ulceration in the left colon (Figure 2B). Pathology revealed mild chronic active colitis. These findings triggered initial treatment with intravenous methylprednisolone 1 mg/kg daily, with good response, and then the patient was discharged with a steroid taper. Neither the PET/CT completed 1 month prior to the colitis episode nor the one completed 1 month after the colitis episode showed any increased FDG uptake in the colon, which is in huge contrast to the endoscopic findings (Figure 3).

## 5. Discussion

The diagnosis and management of colitis during ICI therapy can be challenging due to its nonspecific symptoms and overlap with infectious and other drug-induced etiologies [15]. Currently, the role of imaging techniques such as CT is limited by their low sensitivity, although there is conflicting evidence [9]. PET scans reveal increased FDG uptake in areas of inflammation and could theoretically be used to predict the onset of subclinical IMC before the onset of symptoms. Our study is the first to explore this, with mixed results. Most confirmed IMC cases in our sample had negative PET scans within the month and a half prior to colitis onset. There was no observed difference in the clinical symptoms of or treatments for colitis between patients with no colonic uptake on PET and those with uptake. There were, however, differences in certain outcomes such as hospitalization, ICI resumption, and the resolution of PET findings after treatment between the 2 groups, although larger scale studies are needed to draw more meaningful conclusions [16].

Because irAEs pose an obstacle to effective and uninterrupted cancer treatment, they have received immense attention in the scientific literature in recent years. Substantial efforts are underway to identify predictive biomarkers that can identify patients at risk for irAEs, but there are many difficulties with this line of research [17]. A few biomarkers associated with irAEs have been identified, such as circulating blood counts and specific proteins, but they are nonspecific [17]. In the absence of such specificity for irAEs, these markers are of little value. In the realm of immune-related colitis, most studies focus on the diagnostic rather than the predictive utility of different means of evaluation. While CT imaging has been shown to have a low sensitivity for diagnosing colitis, PET/CT has shown some promise and has been found to be associated with clinical symptoms [5,6,9]. However, it has been overshadowed by simpler and more sensitive tests. Fecal lactoferrin, for instance, was found to have a 90% sensitivity for identifying histological inflammation in patients with suspected IMC, while fecal calprotectin was closely associated with the presence of mucosal ulceration [18,19]. Our study shows that, even in a predictive capacity, PET/CT may not be a reliable instrument, as there is large variation in PET findings and subsequent clinical symptoms. Even if imaging modalities were to have diagnostic or predictive potential, endoscopy remains a more useful tool.

Endoscopic evaluation has been shown to help stratify patients based on their need for more aggressive treatments, thus guiding management [18]. Because of this, imaging, and PET/CT in particular, might have a much greater impact on management in a predictive role—identifying subclinical inflammation, for instance. To date, only two case series, with small sample sizes, have explored this application. Eshghi et al. reported that, of 18 patients receiving immunotherapy who had pre-ICI and follow-up PET/CTs, the 6 who developed immune-mediated thyroiditis had significantly higher levels of glucose uptake on PET/CT at 10 to 16 weeks after ICI initiation than those who did not develop thyroiditis [19,20]. In another study, Hribernik et al. compared the FDG uptake of 6 IMC patients with that of 15 controls with no irAEs. The researchers found that FDG standardized uptake values prior to clinical symptom onset were significantly higher among the IMC patients, with all six patients showing signs of FDG uptake [20,21]. Our study, although limited in design and lacking an adequate control group, found that, of 33 patients with IMC, more than two-thirds had no evidence of FDG uptake in the month before clinical symptom onset. While we are unable to draw meaningful conclusions from this finding, it calls into question the efficacy of PET/CT in predicting the onset of IMC. Ultimately, well-designed retrospective or prospective studies with much larger sample sizes are needed to better answer this question.

The possibility of predicting irAEs could, in theory, greatly impact the way we approach the management of ICI toxicities. More research is needed before that becomes a reality, however. One issue is how meaningful the information truly is. In the realm of colitis specifically, a PET/CT with FDG uptake in the colon in the absence of IMC symptoms may not be clinically relevant. Putting aside PET/CT’s low specificity for IMC, patients with positive PET/CT findings with no clinical symptoms are unlikely to be treated, for the sake of primary prevention, with the standard-of-care immunosuppressive regimens typically used. Patients who do develop symptoms will likely need endoscopic evaluation, especially if those symptoms are moderate to severe, which is frequently the case [2]. Another issue that arises is one of practicality—if PET/CT is not an adequate diagnostic tool, it is unlikely to be an effective predictive tool.

Endoscopic evaluation with biopsy remains the gold standard for the diagnosis and risk stratification of IMC patients [21,22]. Aside from establishing a diagnosis, it provides useful information to help guide management and evaluate responsiveness to treatment. One study found that high-risk endoscopic features such as extensive inflammation and ulcers deeper than 2 mm or larger than 1 cm were associated with the need for SIT [18]. Several studies have shown that certain endoscopic findings, such as ulceration or microscopic subtypes, were associated with colitis responsiveness to corticosteroids and time to treatment failure, respectively [22,23,24,25]. Building on these findings, our group devised a scoring system for various endoscopic features of IMC that had a high specificity for predicting SIT use [13]. We also found that the time to endoscopy was positively correlated with the time to SIT initiation [25]. Interestingly, in that same study, clinical symptoms poorly correlated with the severity of endoscopic inflammation and the need for SIT [25]. Taking these results into consideration, positive findings on PET/CT before or after symptom onset do not seem to contribute meaningfully to the evaluation and management of IMC. Our current study also suggests that PET/CT findings in the colon may not follow the trajectory of clinical symptoms, with almost half of patients having persistent FDG uptake in their colon despite the clinical resolution of their colitis. Given its infeasibility and inconsistency, we believe there is a very limited role for PET/CT in the evaluation, diagnosis, or management of IMC.

There are several limitations to this study. It was a retrospective study with design restrictions due to the overwhelming number of patients available in the database and the limited availability of our radiology team to interpret the tens of thousands of CT scans initially extracted. This necessitated an arbitrary sample of 33 patients, which introduced some bias and prevented us from performing adequate subgroup analyses. The lack of a control group with negative/positive PETs and no IMC further weakened the internal validity of the study. Finally, since this was a retrospective study, we relied on the documentation available in patients’ medical records, which can be incomplete or inaccurate.

## 6. Conclusions

This is the largest study to date exploring the utility of PET/CT scans, routinely used for cancer surveillance, in predicting the onset of IMC and its responsiveness to treatment once it does occur. While descriptive in nature, our findings suggest that PET/CT findings are an unreliable marker for IMC disease activity and should not be relied on to decide on management. However, well-designed retrospective and prospective studies are needed to draw definitive conclusions, and more research into this subject is needed before any conclusions can be drawn.

## Figures and Tables

**Table 1:** Patient Characteristics, N = 33.

Characteristic	No. (%)
Age, years, median (IQR)	62 (52–76)
Sex, female	20 (60.6%)
Race, White	29 (87.9%)
Cancer type	
Melanoma	8 (26.7%)
Lung	14 (46.7%)
Head and neck	3 (10.0%)
Other *	5 (16.7%)
Medical History	
Hypertension	16 (48.5%)
Diabetes Mellitus	3 (9.1%)
End-Stage Renal Disease	0 (0%)
Cirrhosis	0 (0%)
ICI type	
Anti–PD-1/-L1	14 (42.4%)
Anti–CTLA-4	2 (6.1%)
Combination	17 (51.5%)
Duration of ICI treatment, months, median (IQR)	6.5 (2.8–12.2)
Vital status	19 (59.4%)
Duration of follow-up, years, median (IQR)	0.9 (0.3–2.7)

Abbreviations: IQR—interquartile range; ICI—immune checkpoint inhibitor; PD-1/L1—programmed death-1/ligand-1; CTLA-4—cytotoxic T-lymphocyte antigen 4. * Other cancer types included two patients with lymphoma, and one each with chordoma, breast cancer, and cervical cancer.

**Table 2:** Colitis-related characteristics of patients with colitis-positive and colitis-negative initial PET/CT findings (N = 33).

Characteristic	Negative PET/CT (n = 24)	Positive PET/CT (n = 9)
Other irAEs	9 (39.1%)	3 (37.5%)
Days from ICI to IMC, median (IQR)	133 (22–307)	140 (109–186)
Time from initial PET/CT to colitis, days, median (IQR) ^1^	46 (42–58)	39 (37–46)
Highest CTCAE grade for diarrhea		
1	9 (39.1%)	4 (50.0%)
2	7 (30.4%)	2 (25.0%)
3	7 (30.4%)	2 (25.0%)
Highest CTCAE grade for colitis, no. (%) [N = 30]		
1	10 (45.5%)	5 (62.5%)
2	11 (50.0%)	3 (37.5%)
3	1 (4.5%)	0 (0%)
Inflammation found in colonoscopy	7 (29.2%)	5 (55.6%)
Diarrhea/colitis treatment		
Supportive	17 (73.9%)	8 (88.9%)
Corticosteroids	16 (69.6%)	6 (66.7%)
SIT	8 (34.8%)	6 (66.7%)
Days of corticosteroids, median (IQR)	34 (14–55)	31 (14–122)
No. of SIT doses, median (IQR)	1 (1–1.5)	1 (1–2)
Outcome of colitis		
Response/remission	18 (81.8%)	5 (71.4%)
Colitis recurrence after medical treatment	4 (18.2%)	2 (28.6%)
Hospitalization	14 (60.9%)	4 (44.4%)
Time from colitis onset to follow-up PET/CT, days, median (IQR)	85 (44–127)	95 (60–122)
Positive PET after colitis treatment	5 (25.0%)	4 (57.1%)
ICI held for colitis	18 (78.3%)	5 (55.5%)
ICI resumption	10 (43.5%)	5 (62.5%)
Cancer outcome		
Remission/stable disease	10 (43.4%)	5 (55.5%)
Cancer progression	13 (56.6%)	4 (44.4%)
Other irAEs	9 (39.1%)	3 (37.5%)

Abbreviations: PET/CT—positron emission tomography/computed tomography; irAE—immune-related adverse events; IQR—interquartile range; CTCAE—Common Terminology Criteria for Adverse Events; SIT—selective immunosuppressive therapy; ICI—immune checkpoint inhibitor. The base number of patients in some rows may be different than the column total due to the presence of missing cases for some variables. ^1^ A Mann–Whitney U test to compare the distribution of time from PET scan to colitis shows no significant difference between the two groups, *p* = 0.128.

## Data Availability

The data sets generated and/or analyzed during the current study are available from the corresponding author upon reasonable request.
